# Clinical and Immunohistochemical epithelial profile of non-healing chronic traumatic ulcers

**DOI:** 10.4317/medoral.23729

**Published:** 2020-07-19

**Authors:** Gerardo Marcelo Gilligan, René Luis Panico, Cecilia Di Tada, Eduardo David Piemonte, Mabel Noemí Brunotto

**Affiliations:** 1 DDS, PhD, Assistant Professor. Oral Medicine Department, Facultad de Odontología, Universidad Nacional de Córdoba, Argentina; 2 DDS, PhD, Head Professor. Oral Medicine Department, Facultad de Odontología, Universidad Nacional de Córdoba, Argentina; 3 MD. Laboratorio de Inmunohistoquímica, Fundación para el Progreso de la Medicina, Córdoba, Argentina; 4 DDS, PhD, Associate Professor. Oral Medicine Department, Facultad de Odontología, Universidad Nacional de Córdoba, Argentina; 5 PhD, Head Professor. Oral Biology Department, Facultad de Odontología, Universidad Nacional de Córdoba, Argentina

## Abstract

**Background:**

Chronic wounds were previously related to cancer. Chronic Traumatic Ulcers (CTU) are lesions caused by chronic mechanical irritation (CMI) frequently diagnosed in Oral Medicine. Although these conditions may reflect a benign nature, some authors have proposed its relationship with malignant transformation. Currently, there are scarce investigations that evaluate biomarkers within CTU. The aim of this study was to evaluate cell differentiation and proliferation biomarkers patterns of CTU and OSCC through recognized markers such as cytokeratin 19 and Ki67 and correlate it with clinical features of both groups of patients.

**Material and Methods:**

A Cross-sectional study of adult patients (n=79), both sexes, attended at Oral Medicine Department, Facultad de Odontología, Universidad Nacional de Córdoba. The patients were classified into two groups: CTU (n=41), and OSCC (n=38). A subset of specimens were immunolabeled with Ki67 and Ck19.

**Results:**

The population consisted of 51.9% male and 48.1% female, with an average of 57.0 ± 13.9. years (OSCC group) and 60.9 ± 14.9 years (CTU group). OSCC group presented higher scores for both biomarkers (Ki67 and Ck19), but only there were differences statistically significant for Ki67 (*p*=0.032). 25% of non-healing CTU were positive with medium scores of Ck19 and showed an immunohistochemical profile similar to OSCC. The lateral tongue was the most frequent site in both groups.

**Conclusions:**

The altered immunohistochemical pattern found in many specimens of CTU was also observed in OSCC. The tongue border presents physiological conditions that could offer a suitable environment for the development of neoplastic events associated with CMI. Further studies are needed to understand the underlying mechanisms that could link oral non-healing ulcers with early malignant changes.

** Key words:**Ck19, Ki67, Oral Cancer, Chronic Traumatic Ulcer, Chronic Mechanical Irritation.

## Introduction

A century ago, malignant tumors were considered as wounds that do not heal. The micro-tumoral environment shares similar pathogenic features with a chronic wound (1) and chronic inflammation is considered one of the hallmarks of cancer (2). For example, Marjolin´s ulcer is considered as a carcinomatous degeneration of a chronic skin wound of traumatic origin and its malignant transformation is unquestionable among the dermatological field. Chronic inflammation and healing failures described in skin ulcers are etiological features associated with malignant transformation (3).

Non-healing ulcers associated with persistent inflammatory factors have been previously correlated with the development of Oral Squamous Cell Carcinoma (OSCC) (4). Chronic mechanical irritation (CMI) of the oral mucosa could lead to the development of several oral lesions including the so-called Chronic Traumatic Ulcer (CTU). This condition is originated by dental, prosthetic and/or functional factors (5). An animal model of CTU was previously studied suggesting the promoter role of CMI during oral carcinogenesis and the clinical relevance of CMI in patients with subclinical tumor initiation (6). Recently, an Indian group proposed that CTU located on the lateral border of the tongue could be considered an Oral Potentially Malignant Disorder (OPMD) (7). Nevertheless, the relationship between CMI and oral carcinogenesis is still a debaTable topic.

The identification of cellular biomarkers patterns in CTU could allow the recognition of early signs of malignancy and could be helpful in monitoring programs and/or early diagnosis of OSCC. The study of epithelium biomarkers within a chronic-irritation damaged tissue would constitute the first step to analyze the common pathogenic mechanisms among both conditions. Nowadays, there is scarce evidence regarding biomarkers of oral CMI-associated lesions.

In the early stages of OSCC, cytokeratins (Cks) have been proven as biomarkers of cellular differentiation patterns. Cks are intermediate filaments of the cytoskeleton of keratinocytes and recognized markers of stratified epithelial differentiation (8). Changes in the expression of Cks have been widely studied in premalignant and malignant lesions (9). Since Ck19 was found in suprabasal layers of dysplastic lesions, it was suggested as a biomarker of early oral carcinogenesis (10). Cyclin Ki67 is a recognized proliferation biomarker. It is a protein strictly confined to the nucleus with an important role in the regulation of the cell cycle (11). The aim of this study was to evaluate a differentiation and cell proliferation pattern through recognized markers (Ck19 and Ki67) and clinical features of CTU and correlate it to the OSCC obtained pattern.

## Material and Methods

A cross-sectional study of adult patients (n=79), both sexes, spontaneously attended at the Oral Medicine Department, Facultad de Odontología Universidad Nacional de Córdoba, was carried out between 2015 and 2018. All procedures performed in studies involving human participants were in accordance with the ethical standards of the institutional and/or national research committee and with the 1964 Helsinki declaration and its later amendments or comparable ethical standards. This study was approved by the Health Research Ethics Institutional Committee of Facultad de Odontología Protocol N° 11-T and Hospital Cordoba CIEIS (Protocol N°1378).

- Clinical exam

The medical-dental, bio-socio-demographic data, and risk factors for OSCC were recorded in clinical history. Tobacco consumption was classified as never smokers, light smokers (365-100.000- lifetime smoked cigarettes), and heavy smokers (> 100.000-lifetime smoked cigarettes). Alcohol consumption was classified as never drinkers, casual drinkers (up to 3 drinks per week) and drinkers (more than 3 drinks per week). Mate consumption was recorded by anamnesis and classified according to the infusion temperature in no mate consumption, warm, hot or very hot”.

**Figure 1:**
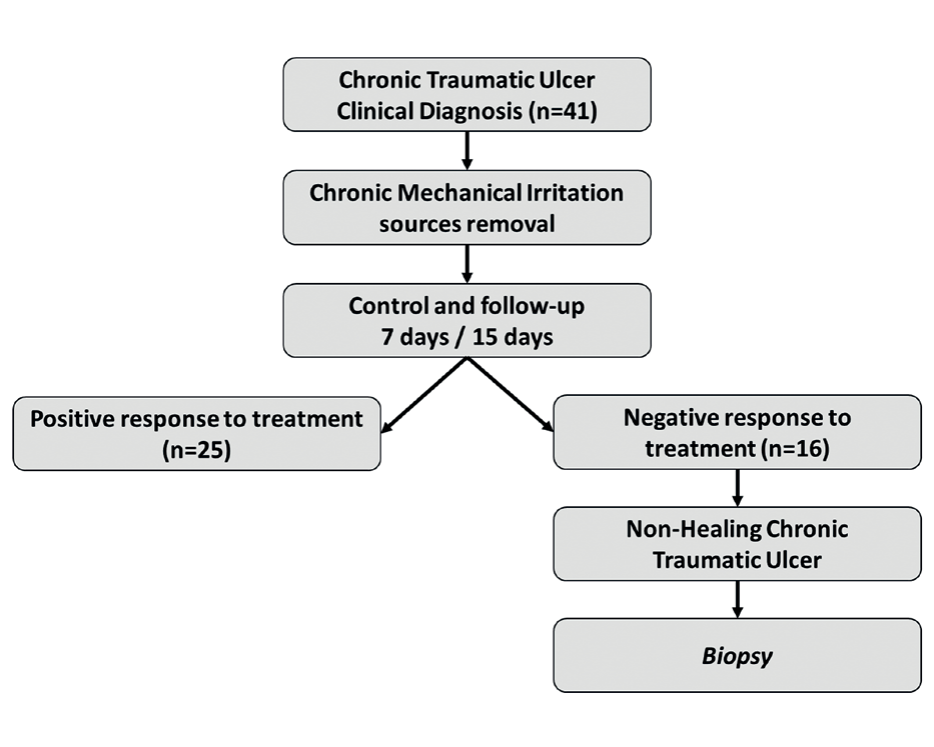
Study Protocol of follow up with the CTU group.

The patients were grouped by clinical-histopathological diagnosis in:

1. CTU: (n=41) according to the criteria of Piemonte *et al*: a) Presence of an objective clinical lesion (CTU) with evolution of over a month. b) The traumatic source must be present before the onset of the lesion (established by anamnesis) c) The traumatic agent must be in direct contact with the lesion, during functional/parafunctional movements or decubitus position (5). Fig. 1 shows the CMI control protocol carried out in the CTU group.

2. OSCC (goal group): (n=38) patients who presented OSCC, In Situ Carcinoma and Verrucous Carcinoma, confirmed by routine anatomopathological examination (ICD-10 code C00.3-C00-6, C01-C06, and D00.0)

Exclusion criteria were treatment with medication such as corticosteroids or chemotherapy drugs, systemic diseases that could modify the clinical behavior of oral lesions, and oral ulcers that could not be clinically associated with a traumatizing agent (i.e. aphthous or infectious ulcers).

Regarding the histopathological criteria, the microscopic observation of a loss of deep epithelium with a fibrin membrane was an essential requirement for the inclusion of the CTU group. Furthermore, they could present one or more of the following histopathological features: epithelial hyperplasia, hyperparakeratosis, acanthosis, and mild dysplasia. On the underlying connective tissue, fibrosis and chronic inflammation could also be observed. The presence of eosinophils was not considered an exclusion criterion. Traumatic Ulcerative Granuloma with Stromal Eosinophilia (TUGSE) is considered by several authors as a non-healing ulcer with a histological presence of eosinophils next to the damaged muscle that could be associated with CMI (12,13). In the clinical context of the CMI criteria used in this study, TUGSE was considered (according to the above cited authors) as a subtype of CTU with a particular feature of eosinophils infiltrate.

- Immunolabeling

Immunostaining of Ki67 and Ck19 was performed in a subset of 16 NHCTU and 18 OSCC. Samples of 4 µm were picked up from paraffin blocks of biopsied specimens. The sections were dewaxed, dehydrated and washed with distilled water. The immunolabeling was performed by peroxidase-antiperoxidase and avidin-biotin technique following the manufacturer's protocol. The following primary antibodies were used: concentrated rabbit anti-human Ki67 (CellMarque, SIGMA ALDRICH, USA), and concentrated monoclonal mouse anti-human Ck19 (CellMarque, SIGMA ALDRICH, USA). The results were considered positive when a brown color appeared in the cell cytoplasm for Ck19 and nucleus for Ki67. All samples were countercoloured with Harris hematoxylin.

The immunolabeling was categorized as negative (score=0): 0 % positive cells; low (score=1): <10% of positive cells, medium (score=2): 10–29% positive cells, and strong/high (score=3):≥ 30% of positive cells, according to Vasca *et al* criteria (14).

- Statistical analysis

The data was described by statistical parameters as average/median, standard error/Rank for quantitative variables, and relative or absolute frequencies for qualitative variables. Chi-Square and Fisher test evaluated the association between groups and estimated Odd Ratio (OR) with 95% Confidence Interval (95%CI). For all test, *p-value* <0.05 was set for statistical significance. Data were analyzed with the Infostat software 2018 professional version and R-software (www.r-project.org).

## Results

- General characteristics

The population consisted of 51.9% (41/79) males and 48.1% (38/79) females (Table 1), with an average age of 57.0 ± 13.9 years for the OSCC group and an average of 60.9 ± 14.9 years for the CTU group (*p* = 0.1546).

**Table 1:**
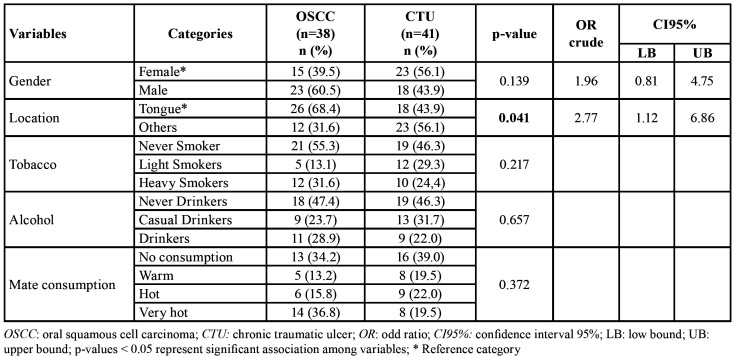
Demographic, risk factors and clinical features of the population under study.

OSCC (n=38) and CTU (n=41) groups showed similar characteristics concerning risk factors such as tobacco, alcohol and mate consumption. The subsite location of oral lesions showed a significantly higher frequency in the lateral border of the tongue in patients in the OSCC group (OR = 2.77, CI95% [1.12, 6.86], *p-value* = 0.0412) (Table 1). In addition, 67% of OSCC patients, were diagnosed when the tumor size was less than 4 cm: Tis(13,16%), T1(21,05%) and T2(34,21%) by TNM classification.

There were analyzed 41 CTU of which 25 showed complete healing after CMI removal. The remaining 16 Non-Healing CTU (NHCTU), mostly located on the lateral tongue border (61%), were biopsied to rule out OSCC. In relation to the presence of CMI associated with OSCC, only 18.4% (7/38) of the patients did not present CMI during the oral examination. Among patients who presented CMI factors, prosthetic or removable factors were more frequent in the CTU group, while fixed or dental factors, functional factors, and combined factors were more frequent in the OSCC group. For each source of CMI, the statistical significance was minor than 0.05, except for combined factors that showed a *p-value* close to the statistical significance (*p* = 0.052). The comparison of the frequency for each CMI factor between Healing CTU (HCTU) and NHCTU, repeated the previous pattern of CMI (CTU vs. OSCC), while when comparing NHCTU with OSCC there were no differences in the frequency pattern (Table 2).

Table 3 shows the main histological features observed in the NHCTU group.

**Table 2:**
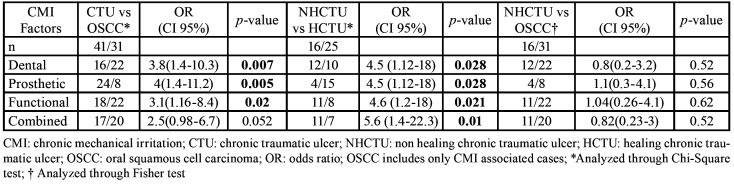
Comparison of CMI factors.

**Table 3:**
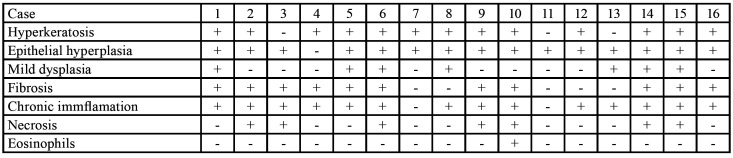
Main histopathological findings in Non-Healing Chronic Traumatic Ulcers.

- Immunolabeling:

OSCC group presented higher scores for both biomarkers (Ki67 and Ck19), but only there were differences statistically significant for Ki67 (X2, *p*=0.032). Table 4 shows the different scores of immunostaining for each biomarker found in both groups of study.

Most of the cases which showed medium and high scores of Ki67-expression were also Ck19 positive (9/12 OSCC samples with medium and high Ki67 scores, showed medium/high Ck19 scores; 3/3 NHCTU samples with medium Ki67 scores, showed medium scores of Ck19 with a suprabasal pattern).

In concordance with the most prevalent location, the lateral border of the tongue was also the subsite with the most presence of positive cells of immunolabelling between both groups. 70% of OSCC specimens with medium and high scores of both markers were located on the lateral tongue border. Verrucous carcinomas were negative for Ck19 expression regardless of the subsite of location.

**Figure 2:**
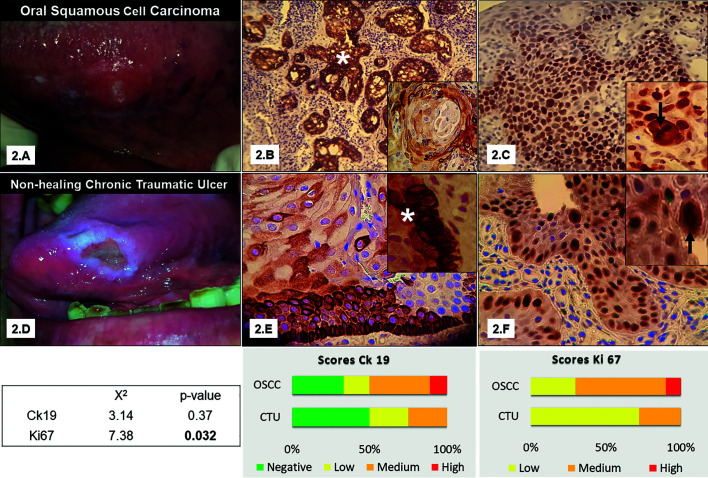
Immunolabeling of Ck19 and Ki67 for Non-healing CTU and OSCC group. The black arrow indicates positive immunolabeling of nuclei and white asterisk indicates positive immunolabeling of cytoplasm. The immunostaining was considered negative when observed 0–5% positive cells. The analysis of the samples was performed by a single-blind examiner. The positive immunostaining was counted for each group and expressed. Stacked bars represent the percentage of immunolabelled samples for each biomarker used according to their score (negative low-medium-high). The sections were analyzed by optical microscopy at a magnification of 20X and 400 X. *p* <0.05 indicates statistical significance. 2A- OSCC of the lateral tongue diagnosed at an early stage. 2B- Islet cells with Ck19 positive cytoplasm. OSCC group. 2C-High index of Ki67, OSCC group. 2D- Non healing Chronic Traumatic Ulcer located on the lateral tongue associated with sharp cups and parafunctional habits (tongue interposition). The ulcer did not heal after 15 days of the CMI source control. 2E Niches of Ck19 positive cells with a suprabasal pattern found within the hyperplastic epithelium of the edges of the NHCTU. 2F Suprabasal expression of Ki67, NHCTU group. Nuclear alterations were evidenced with the Ki67 immunostaining.

NHCTU located on the lateral tongue were associated with a mixed source of CMI, with a prevalence of dental (fixed) and functional factors with medium scores of Ki67/Ck19. NHCTU located on the vestibular sulcus (associated with prosthetic trauma), showed negative Ck19 scores and low Ki67 scores. There was no relationship between other risk factors such as tobacco, alcohol and mate consumption, histological features, and the immunoexpression scores. Fig. 2 illustrates the different patterns and scores of immunolabelling among both groups and their clinical presentation.

**Table 4:**
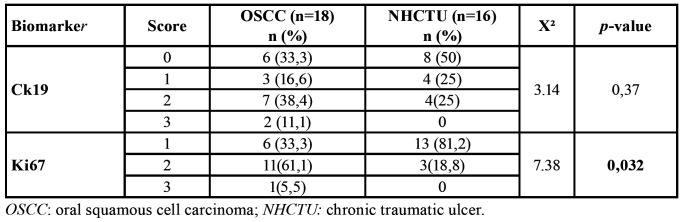
Immunolabelling for Ck19 and Ki67.

## Discussion

Our results showed that a subset of NHCTU, mainly located on the lateral border of the tongue, showed a suprabasal pattern of Ck19, which was not described before. Furthermore, these features, usually associated to early oral carcinogenesis, were found in NHCTU. Cks were widely studied in the field of OSCC and OPMD. Ck19 is eventually found in the basal layer of the normal oral epithelium of non-keratinized mucosa, but overexpressed in OSCC (15), OPMD, and epithelial dysplasia (16). Ck19 was also proposed as a nonspecific Oral Keratinocytes Stem Cells (OKSC) marker (17). Kale *et al*, found a suprabasal expression of Ck19 in normal oral mucosa adjacent to OSCC suggesting that this suprabasal expression never occurs in non-malignant tissue and depicts not only a simple hyperproliferative status but also a marker associated with premalignancy (18). However, Ck19 observed in NHCTU could be indicative of an immature epithelium related to unsuccessful healing mechanisms and could not be associated specifically with malignancy. A chronic injury leads to a healing process that involves the migration and the development of a hyperproliferative environment of OKSC next to the edges of the wound. This process requires tightly controlled mechanisms that balance epithelial cell production and loss (19). Epithelial immature cells, such as those normally found during renewal, are considered the target of carcinogens that could lead to accumulation of genetic changes (20).

Many studies highlighted an increase of Ki67 expression in dysplastic lesions (21) and its association with the clinical severity of the disease (22). The persistent expression of suprabasal Ki67 could be represented by cellular subclones with an invasive ability and the consequent development of a precancerous tumorigenic niche (23). The increase of cell proliferation due to CMI was previously proposed as a predisposing event in a multistep model of oral carcinogenesis (5). NHCTU would meet those requirements and could act as a promoter cofactor in malignant transformation (24). 

Regarding ethical concerns, the natural story of human CTU cannot be entirety studied due to the need to apply therapeutic protocols. Control of traumatic sources prior to diagnosis biopsy could have partially modified the proliferation patterns of NHCTU. In consequence, the obtained patterns in those cases, may not reflect the genuine scenario at the time of the first visit. Regarding this, a suprabasal Ki67 pattern was observed in a subset of NHCTU. However, the persistence of these markers in NHCTU, even after having eliminated traumatizing agents, could be associated with exaggerated mechanisms of re-epithelialization in response to an unresolved traumatic wound.

CMI-associated OSCC and NHCTU were predominantly located on the lateral border of the tongue, and mainly related to dental and functional CMI factors. On the other hand, it was observed negative or low scores among CTU located on the buccal mucosa or the vestibular sulcus and related to prosthetic CMI. The location and the source of CMI could condition the different pathways of tissue damage or healing process. OSCC arises most frequently at high-risk areas such as the ventrolateral tongue and the floor of the mouth. The lateral border of the tongue shows a non-keratinized epithelium that could facilitate the penetration of other carcinogens (25) as well as the most proliferative rates among other subsites of the oral mucosa (26). This area was previously described as a transitional zone (TZ) between the epithelium of the dorsum and the ventral tongue (27). TZs are considered hazardous subsites for cancer development because they harbor stem cells-niches (with a permanent autorenewal ability) (28). That stem cells-niche could be the target of oxidative stress originated from chronic inflammation, chemical carcinogens, etc (29). In this context, a genuine per se replicative status of the ventrolateral tongue and its anatomical adjacency to different CMI sources could increase the risk and the frequency of OSCC in this area.

**Figure 3:**
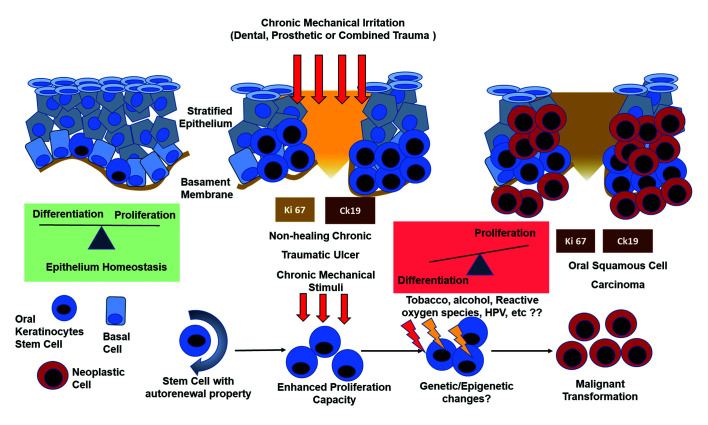
The epithelium homeostasis of the oral mucosa involves a balance between cell differentiation and cell proliferation. CMI could generate a stimulus in the increase of immature Ck19 cells (OKSC) and a continuous presence of these cells, known for their genomic instability, in the suprabasal layers of the oral epithelium. These cells, considered as a target of different carcinogens, are also involved in the self-renewal of the epithelium and healing events. An epithelial environment of hyperproliferation and cellular immaturity is generated chronically next to a wound with no tendency to cure. CMI could act as a promoter of oral carcinogenesis through these supposed mechanisms.

Oral carcinogenesis is a multifactorial and multistep process that involves initiation, promotion and tumoral progression. CMI could act as a local stimulus for a persistent hyperproliferative status of immature cells, characterized by its vulnerability to the genetic damage. Thus, CMI could be considered not only a promoter but also a conditioning cofactor of the initiation phase. Fig. 3 shows a summary of the results concerning the biomarkers immunoexpression. In addition, it hypothesizes possible changes associated with CMI in the context of the multifactorial carcinogenesis.

Our results show similarities between HNCTU and OSCC, suggesting that a subset of both conditions could show an evolutive correlation. Essentially, the present study only proves that several NHCTU showed a risky scenario related to a chronic hyperproliferative and immature status. Nevertheless, there are key topics to be answered regarding mild dysplasia in NHCTU. If those linger on for months or years, could that reactive changes become a “true” dysplasia? More strong evidence should be generated using other biomarkers and/or different research approaches.

Conclusion

Chronic wounds were historically related to cancer but unresolved ulcers of the oral cavity, still lack sufficient evidence to accept it as a condition with potential malignant transformation. A group of non-healing CTU harbored immunohistochemical patterns of cell differentiation and proliferation similar to those observed in OSCC and described in previous OPMD studies. These findings could be useful to start proper researches to increase the evidence about this issue. Further studies are needed to improve the understanding of the underlying mechanisms observed in NHCTU and its relationship with oral carcinogenesis.
